# Prediagnostic opioid use and survival in multiple myeloma: a nationwide register-based study

**DOI:** 10.1038/s41598-026-59999-2

**Published:** 2026-07-09

**Authors:** Jojo Biel-Nielsen Dietz, Ragnar Pétur Kristjánsson, Ólafur Birgir Davídsson, Klaus Rostgaard, Tereza Fait Kadlec, Emelie Curovic Rotbain, Signe Holst Søegaard, Sigrún Thorsteinsdóttir, Agoston Gyula Szabo, Carsten Utoft Niemann, Henrik Hjalgrim, Christian Brieghel

**Affiliations:** 1 Hematology, Danish Cancer Society Research Center, Copenhagen, Denmark; 2 Department of Epidemiology Research, Statens Serum Institute, Copenhagen, Denmark; 3 Department of Hematology, Rigshospitalet, University Hospital of Copenhagen, Copenhagen, Denmark; 4 Department of Congenital Disorders, Statens Serum Institute, Copenhagen, Denmark; 5 Department of Clinical Medicine, University of Copenhagen, Copenhagen, Denmark

**Keywords:** Multiple myeloma, Opioid use, Prognosis, Overall survival, Cancer, Diseases, Medical research, Oncology

## Abstract

Multiple myeloma (MM) accounts for approximately 1% of incident cancers worldwide and carries substantial mortality. Diagnostic delay is common, with more than one-third of patients requiring emergency care at diagnosis, a presentation linked to inferior prognosis. Pain is a frequent early MM symptom, yet the extent and prognostic relevance of prediagnostic opioid use remains poorly characterized. Using nationwide Danish registers, we conducted a matched case-control analysis of prediagnostic opioid use, irrespective of indication, and its association with overall survival (OS) among MM patients. The study included 6,953 MM patients diagnosed in 2001–2021 and 101,793 matched population controls. Compared with controls, opioid use was first elevated among MM cases approximately 2.25 years before diagnosis, increasing more consistently closer to diagnosis. High prediagnostic opioid use (≥ 10 mg daily oral morphine equivalents) was associated with shorter OS, compared with no opioid use (1-year OS 72% vs. 82%; adjusted hazard ratio 1.23, 95% CI 1.07–1.41). These findings indicate that opioid use increases closer to MM diagnosis date, likely reflecting pain related to undiagnosed MM. Furthermore, prediagnostic opioid use was associated with shorter OS independently of available prognostic factors, including ISS stage and myeloma bone disease.

**Supplementary Information:**

The online version contains supplementary material available at https://doi.org/10.1038/s41598-026-59999-2.

## Introduction

Multiple myeloma (MM) is a common hematological cancer, accounting for approximately 1% of all new cancer cases worldwide each year^1^. MM is preceded by two asymptomatic precursor conditions, monoclonal gammopathy of undetermined significance (MGUS) and smoldering MM (SMM), with yearly risks of 1–2% and 10%, respectively, of developing into overt symptomatic MM within the following five years^2,3^. Although almost all MM cases evolve from MGUS^4^, more than 95% of patients are diagnosed with MM without prior clinical identification of MGUS or SMM^5^.

More than half of patients later diagnosed with MM initially present to a general practitioner^6^, but the nonspecific nature of early symptoms makes clinical recognition in primary care notoriously difficult^7,8^. Approximately two-thirds of patients diagnosed with MM present with back pain attributable to myeloma bone disease (MBD)^9^, and a similar proportion report back pain to their general practitioner in the two years leading up to their diagnosis^10^. These observations indicate missed diagnostic opportunities in a window spanning multiple years, which is particularly concerning as 37% of patients with MM require emergency care at diagnosis, a presentation associated with a poorer prognosis^11^.

Therapeutic options for MM have significantly advanced over the past 20 years, leading to improved survival after diagnosis^12^. However, the improved outcomes rely in part on early treatment initiation^12^, underscoring the importance of timely diagnosis. Diagnostic delay may also result in severe organ damage and complications such as spinal cord compression due to MBD, irreversible renal injury, and severe infections before treatment can be initiated^13^. Avoiding diagnostic delay is therefore crucial.

In a patient group for whom bone pain is a major symptom, an increased rate of analgesic use prior to diagnosis can be hypothesized. A study by Howell et al. has highlighted a median delay of up to 5 months between the onset of symptoms and the diagnosis of MM^8^, indicative of a prolonged period during which the use of analgesics, including opioids, may be significantly increased.

Despite these observations, prediagnostic opioid use in MM and its potential prognostic significance remain largely uncharacterized. Therefore, we aimed to characterize opioid use prior to MM diagnosis in a population-wide setting, comparing the opioid use patterns of patients later diagnosed with MM with those of a matched control group. Furthermore, we aimed to assess the association between opioid use before MM diagnosis and overall survival (OS) after diagnosis in patients with MM.

## Results

### Characteristics of the study population

The study cohort comprised 6,953 patients diagnosed with MM in Denmark between 2001 and 2021 and 101,793 matched population controls selected from the Danish Civil Registration System, as described in the Methods. Overall, 56% of individuals were male and 44% were female. The mean age at diagnosis of MM was 69 years (standard deviations [SD] ± 11), with a mean follow-up time of 4.2 (± 3.8) years after diagnosis among MM cases. Data on International Staging System (ISS) stage and MBD at diagnosis were available for 79% (*n* = 5,494) of the patients with MM. Within this subset, 65.5% (*n* = 3,599) had MBD at diagnosis, and the distribution across ISS stage at diagnosis was 34.5% (*n* = 1,896), 37.6% (*n* = 2,066), and 27.9% (*n* = 1,532) at stage I, II, and III, respectively (Table 1).

**Table 1:** Descriptive characteristics of the study population.

	MM cases (*n* = 6,953)	Matched controls (*n* = 101,793)
Sex, *n* (%)		
Male	3,902 (56.1%)	57,011 (56.0%)
Female	3,051 (43.9%)	44,782 (44.0%)
Age at index date (years)		
Mean (± SD)	69.3 (± 11.3)	69.1 (± 11.2)
Follow-up (years)		
Mean (± SD)	4.2 (± 3.8)	-
MBD at diagnosis (*n* = 5,494)		
Yes	3,599 (65.5%)	-
No	1,895 (34.5%)	-
ISS stage at diagnosis (*n* = 5,494)		
I	1,896 (34.5%)	-
II	2,066 (37.6%)	-
III	1,532 (27.9%)	-

ISS: International Staging System, MBD: Myeloma bone disease, MM: Multiple Myeloma, SD: Standard Deviation.

### Prediagnostic opioid use and multiple myeloma diagnosis

Across the five years preceding the index date, defined as the date of MM diagnosis for cases and the corresponding matched date for controls, redemption of ≥ 1 prescription for any opioid was consistently more common among MM cases than matched controls. In the year before the index date, 43.3% of cases and 12.3% of controls redeemed ≥ 1 prescription for any opioid. In the periods of 1–2, 2–3, 3–4, and 4–5 years before the index date, the corresponding proportions were 15.0%, 12.6%, 11.4%, and 10.4% among cases, compared with 11.7%, 10.4%, 10.4%, and 9.9% among controls (Table 2).

**Table 2:** Opioid use in the study population.

	MM cases (*n* = 6,953)	Matched controls (*n* = 101,793)	*P* ^1^
Individuals receiving ≥ 1 opioid prescription in the 5 years before the index date, *n* (%)^2^			
0–1 year before index date	3,012 (43.3%)	12,530 (12.3%)	
1–2 years before index date	1,046 (15.0%)	11,920 (11.7%)	
2–3 years before index date	875 (12.6%)	10,643 (10.4%)	
3–4 years before index date	793 (11.4%)	10,643 (10.4%)	
4–5 years before index date	723 (10.4%)	10,051 (9.9%)	
Average daily OME during the 3–12 months before the index date, *n* (%)			
0 mg	5,418 (77.9%)	89,471 (87.9%)	< 0.001
<10 mg	1,128 (16.2%)	8,550 (8.4%)	
≥10 mg	407 (5.9%)	3,772 (3.7%)	

MM: Multiple Myeloma, OME: Oral Morphine Equivalents.

^1^Pearson’s chi-square test.

^2^Same individuals may appear in more than one category.

Opioid use in the 3–12 months before the index date was quantified as average daily oral morphine equivalents (OME). The distribution of average daily OME categories differed significantly between MM cases and controls (Pearson’s chi-square test, *P* < 0.001), with opioid use being more prevalent among cases than controls: 16.2% (*n* = 1,128) of cases versus 8.4% (*n* = 8,550) of controls had low use (< 10 mg OME/day), and 5.9% (*n* = 407) versus 3.7% (*n* = 3,772) had high use (≥ 10 mg OME/day) (Table 2).

**Fig. 1:**
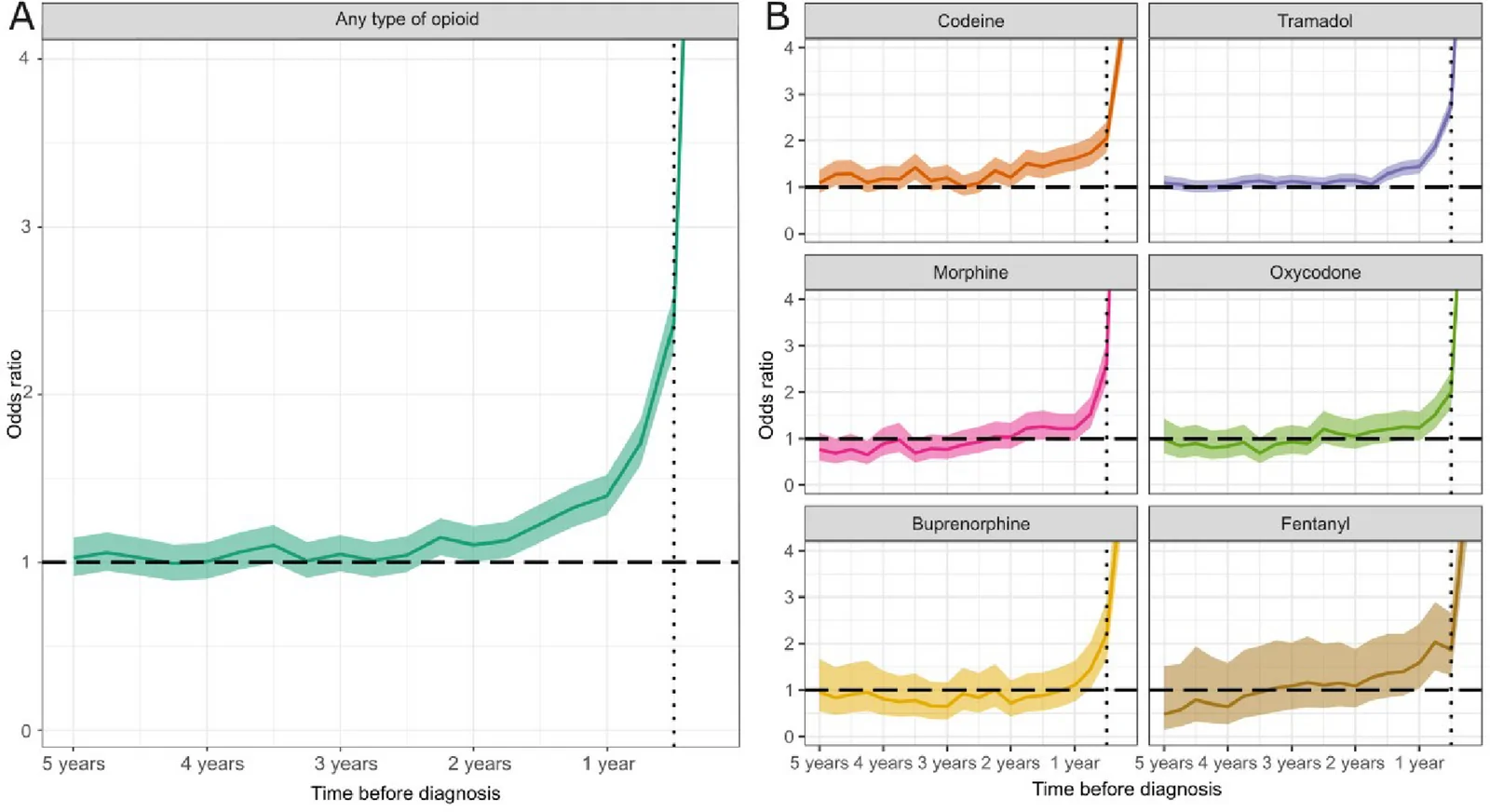
Odds ratios (ORs) for later diagnosis of multiple myeloma associated with opioid prescription redemption in each 3-month interval during the 5 years before the index date among cases (*n* = 6,953) compared with matched controls (*n* = 101,793). (**A**) Any opioid; (**B**) Specific opioids. The vertical dotted line marks 6 months before the index date.

Figure 1 shows odds ratios (ORs) for MM diagnosis associated with redemption of an opioid prescription in consecutive 3-month intervals during the five years before the index date. The final 3-month pre-index interval was excluded because it likely reflects opioids prescribed in relation to the diagnostic evaluation. Compared with matched controls, the OR for MM was first statistically elevated approximately 2.25 years before the index date (OR 1.15, 95% CI 1.04–1.22, false discovery rate [FDR]-adjusted *P* = 0.01), with a more consistent increase closer to diagnosis, reaching 2.42 (95% CI 2.25–2.60, FDR-adjusted *P* < 0.001) in the last included interval (3–6 months pre-index). When stratified by opioid type, the timing of statistically significant associations differed by opioid potency: for lower-potency opioids (codeine and tramadol), ORs were increased from approximately 2.25 years pre-index, whereas for higher-potency opioids (buprenorphine and fentanyl) ORs increased primarily during the year immediately preceding the index date. Quarterly ORs and FDR-adjusted P-values by opioid category are provided in Supplementary Table 1. In homogeneity analyses using Breslow-Day tests to compare adjacent crude interval-specific ORs for any opioid use, statistically significant heterogeneity was first observed between 1.00 and 0.75 years before the index date (Supplementary Table 2). These analyses supported a more pronounced increase in opioid use during the year preceding MM diagnosis.

### Prediagnostic opioid use and survival after diagnosis

Among patients diagnosed with MM (*n* = 6,953), OS was examined according to average daily OME in the 3–12 months before diagnosis.

**Fig. 2:**
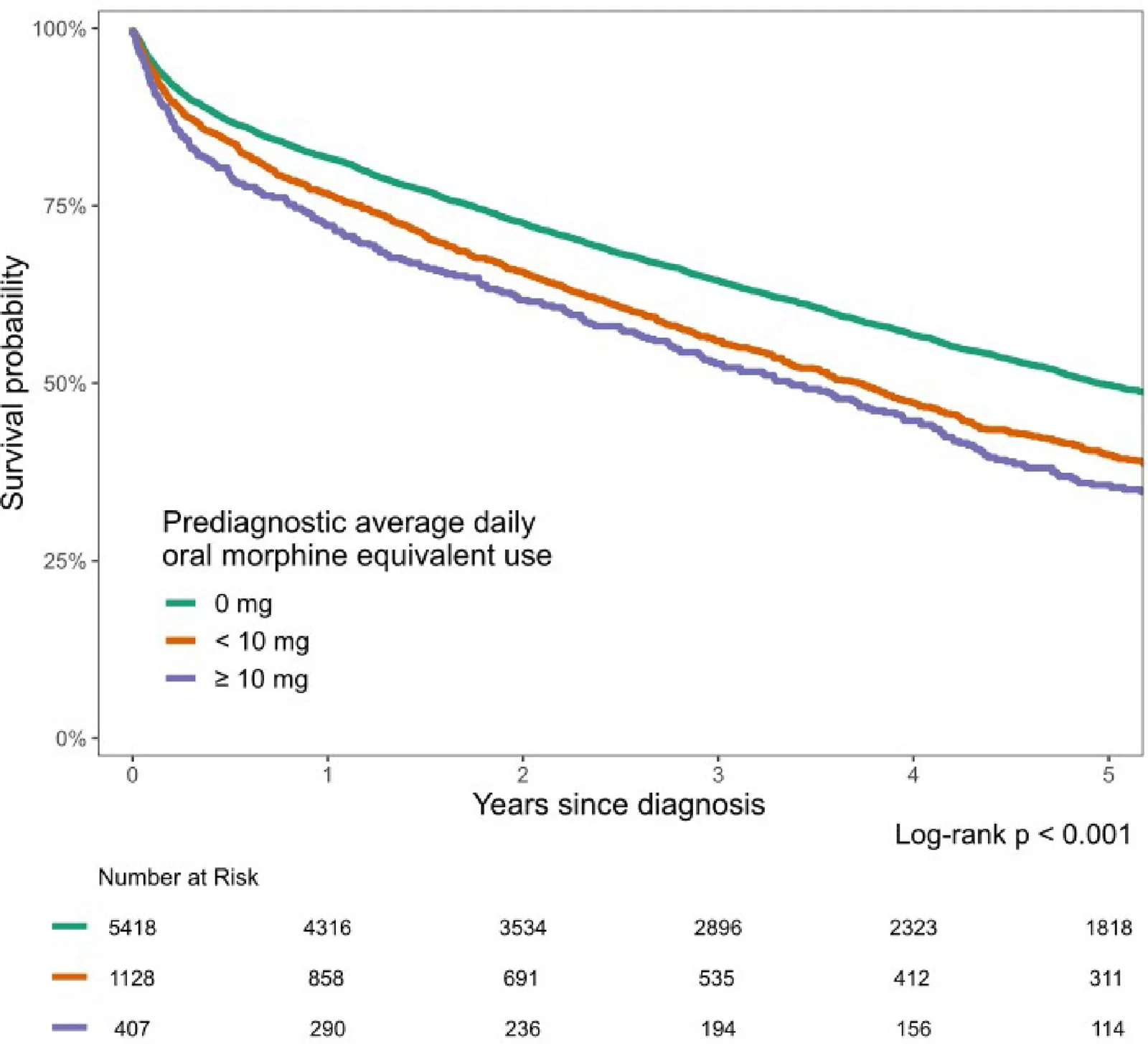
Overall survival of patients diagnosed with multiple myeloma stratified by average daily oral morphine equivalent (OME) use in the 3–12 months before diagnosis (*n* = 6,953). The overall log-rank test comparing all three groups was significant (*P* < 0.001). Pairwise log-rank tests showed significantly lower OS for < 10 mg/day and ≥ 10 mg/day compared with no opioid use with both comparisons reaching *P* < 0.001, while OS did not differ significantly between < 10 mg/day and ≥ 10 mg/day (*P* = 0.087).

The Kaplan-Meier curves stratified by average daily OME in the 3–12 months before MM diagnosis, are presented in Fig. 2 and Supplementary Table 3a. Higher prediagnostic OME category was associated with lower OS (log-rank *P* < 0.001). In pairwise log-rank tests, OS was significantly lower among patients with < 10 mg/day and ≥ 10 mg/day compared with patients with no opioid use, with both comparisons reaching *P* < 0.001, whereas OS did not differ significantly between the < 10 mg/day and ≥ 10 mg/day groups (*P* = 0.087; Supplementary Table 3b).

At 1 year post-diagnosis, survival was 82% (95% CI 81–83%) among patients with no opioid use, compared with 77% (95% CI 74–79%) for < 10 mg and 72% (95% CI 68–77%) for ≥ 10 mg (log-rank *P* < 0.001; Supplementary Table 3a).

Average daily OME in the 3–12 months before MM diagnosis ≥ 10 mg was significantly associated with increased hazard ratio (HR) for death after diagnosis (HR 1.51, 95% CI 1.32–1.73). After adjusting for sex, age at diagnosis, region of residence, calendar year of diagnosis, ISS stage, and presence of MBD at diagnosis, higher average daily OME in the 3–12 months before MM diagnosis remained independently associated with increased HR for death (HR 1.23, 95% CI 1.07–1.41 [Table 3]).

**Table 3:** Crude and adjusted hazard ratios for death with 95% confidence intervals associated with average opioid use in the 3–12 months before multiple myeloma diagnosis (*n* = 5,494).

		Univariable analysis		Multivariable analysis	
	*N*	Crude HR (95% CI)	Crude *P*	Adjusted HR (95% CI)^1^	Adjusted *P*
Sex					
Female	2,422	*Ref*		*Ref*	
Male	3,072	1.15 (1.08, 1.24)	< 0.001	1.23 (1.15, 1.32)	< 0.001
Age	5,494	1.06 (1.05, 1.06)	< 0.001	1.06 (1.06, 1.07)	< 0.001
ISS stage					
I	1,896	*Ref*		*Ref*	
II	2,066	1.81 (1.65, 1.97)	< 0.001	1.60 (1.46, 1.75)	< 0.001
III	1,532	2.72 (2.48, 2.98)	< 0.001	2.39 (2.17, 2.62)	< 0.001
MBD					
No	1,895	*Ref*		*Ref*	
Yes	3,599	1.37 (1.26, 1.48)	< 0.001	1.31 (1.21, 1.42)	< 0.001
Average daily OME during the 3–12 months before MM diagnosis					
0 mg	4,262	*Ref*		*Ref*	
< 10 mg	925	1.30 (1.19, 1.42)	< 0.001	1.19 (1.09, 1.31)	< 0.001
≥ 10 mg	307	1.51 (1.32, 1.73)	< 0.001	1.23 (1.07, 1.41)	0.003

CI: Confidence Interval, HR: Hazard Ratio, ISS: International Staging System, MBD: Myeloma Bone Disease, MM: Multiple Myeloma, OME: Oral Morphine Equivalents.

^1^Adjusted for all other factors presented in the table, as well as calendar year at diagnosis and region of residence.

## Discussion

In this retrospective register-based case-control study we investigated opioid use for any indication in the 5 years preceding date of MM diagnosis and its impact on OS in MM patients. We observed significantly increased use of opioids among MM patients compared to controls up to 2.25 years before the index date. Moreover, we observed that prediagnostic opioid use, irrespective of indication, was associated with a significantly shorter OS after MM diagnosis.

The increased opioid use observed may represent attempts to manage pain resulting from undiagnosed MM, particularly since bone pain is one of the most frequently reported symptoms by MM patients^14,15^. Moreover, MM’s precursor condition MGUS^2^ has been associated with an increased risk of bone fractures^4,16–19^, which may partly contribute to the elevated opioid use observed prior to MM diagnosis. However, increased opioid use before diagnosis is not specific to MM. In a population-based Australian study of patients initiating opioids before cancer diagnosis, opioid dispensing commonly increased around the time of diagnosis across cancer types, and opioid use patterns were heterogeneous and partly related to non-cancer factors^20^.

The past few decades have seen the discovery and implementation of novel and better-tolerated MM treatments that can be used earlier in the disease course, such as CD38 antibodies, CAR-T cell therapy and bispecific antibodies^12,21–24^. Therefore, the early detection of MM has become increasingly important to reduce both morbidity and mortality. Whilst back pain may be an early clinical indication of MM, general practitioners may rightfully not suspect MM considering that only a small fraction of back pain cases in general practice are later attributed to malignancy^25^. This diagnostic challenge is reflected by previous reports showing that more than half of MM patients consult their general practitioner three or more times before hospital referral^26^. In the present study, opioid use was increased before MM diagnosis, particularly close to the index date. Although prescription indications were unavailable, this temporal pattern is compatible with pain related to undiagnosed MM and aligns with previous reports of diagnostic delay.

We also observed an association between prediagnostic opioid use and reduced OS. While this might be due to more advanced disease stage or the presence of MBD, opioid use remained associated with reduced OS even after adjusting for disease stage via ISS and MBD at diagnosis. Presumably, prediagnostic opioid use represents naïve symptom management, and may indicate increased frailty, or the presence of other indicators of poor prognosis rather than a direct effect of opioids on survival^27,28^. Frailty in MM patients previously classified using different indices, such as the patient-reported frailty phenotype or International Myeloma working group frailty index^27,29^, is a well-known prognostic factor, conferring decreased survival and worsened prognosis. Efforts to refine and individualize MM prognostication for precision medicine could thus incorporate clinical factors such as performance status, frailty, and opioid use, some of which have already been suggested by others^15^. These aspects are neither included in the ISS, the revised ISS (R-ISS), nor in the second revised ISS (R2-ISS)^30,31^.

This study’s strengths include its large sample size and the use of nationwide register data, allowing for a comprehensive examination of opioid use patterns before MM and the impact on MM prognosis. The study’s limitations include that information on the indications for opioid prescriptions was not available. In addition, the survival analyses did not include frailty, or anti-myeloma treatment. These factors may partly explain the association between prediagnostic opioid use and OS, as they are likely related to both opioid use before diagnosis and prognosis after diagnosis. Furthermore, it was not possible to distinguish between MM and SMM in the Danish Cancer Register, and we thus cannot identify and exclude patients with SMM. Finally, the long study period may have encompassed changes in pain management, opioid prescribing, and MM diagnostic practice over time, although, calendar year was included in the regression models to partly account for temporal changes.

## Conclusions

In this retrospective register-based case-control study, opioid use was increased among individuals later diagnosed with MM, particularly closer to the index date. This temporal pattern may reflect pain related to undiagnosed MM and is consistent with previous reports of diagnostic delay. Additionally, prediagnostic opioid use was associated with shorter OS after diagnosis, independently of available prognostic factors, including ISS stage and myeloma bone disease.

## Methods

### Data sources

This nationwide register-based study was conducted in Denmark. Individual-level data were linked across registers using the unique personal identification number assigned to all residents. Cancer diagnoses and dates of diagnosis were obtained from the Danish Cancer Register, which holds information on all individuals diagnosed with cancer in Denmark since 1943^32^. Demographic information including sex, date of birth, current and past residence, as well as dates of emigration and death were obtained from the Danish Civil Registration System^33,34^. Redeemed outpatient prescriptions, including dispensing date and Anatomical Therapeutic Chemical (ATC) classification, strength, administration route and pack size were obtained from the Danish National Prescription Register^35^. Because prescriptions for children were recorded under a guardian’s identifier before 1996, analyses of prescription data were restricted to dispensations from January 1, 1996 onwards. Clinical information at diagnosis, including ISS stage and MBD, was obtained from the Danish National Multiple Myeloma Register, which holds information on all patients diagnosed with MM, SMM, solitary plasmacytomas, and plasma cell leukemia in Denmark since 2005^36^.

### Study population

We identified all individuals diagnosed with MM in Denmark during January 1, 2001 to December 31, 2021 in the Danish Cancer Register (ICD-10 C90.0). For each case, the index date was defined as the date of MM diagnosis. Using incidence-density sampling from the Danish Civil Registration System, we randomly selected 5–15 population controls matched to each case on sex and year of birth. Controls were sampled from the population at risk at each index date, required to be alive and residing in Denmark at that date, and assigned the index date of their matched case. Controls could later be diagnosed with MM and could be sampled more than once^37^. Because eligible controls were readily available in the nationwide population register, up to 15 controls per case were used to improve precision.

Individuals were excluded if they were < 18 years of age at the index date or had a diagnosis of any cancer other than non-melanoma skin cancer before the index date.

**Fig. 3:**
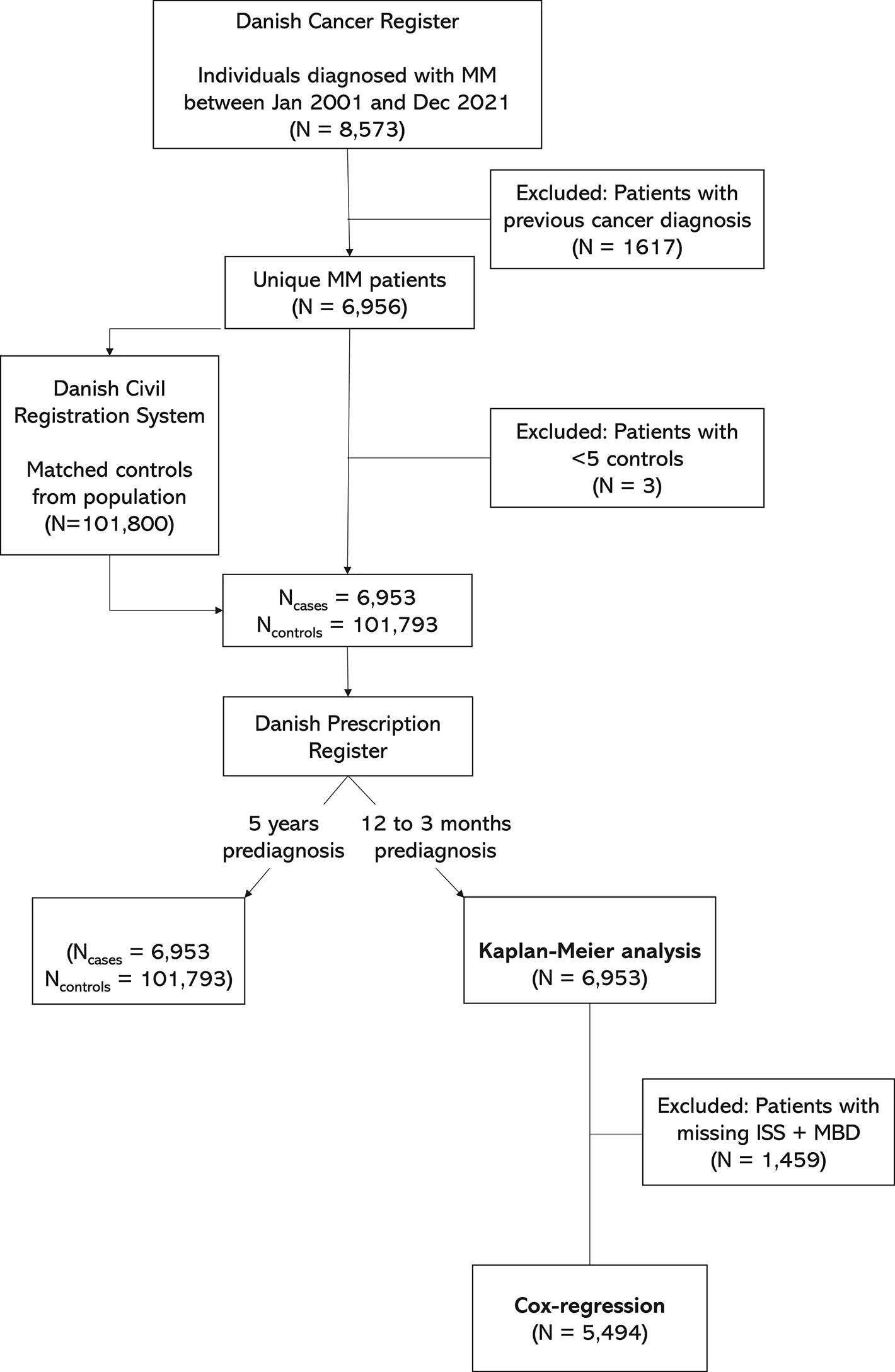
Flow diagram depicting cohort construction for the case-control and survival analyses. MM: Multiple myeloma, ISS: International Staging System, MBD: Myeloma Bone Disease.

Overall survival analyses were conducted among MM cases (*n* = 6,953) in relation to average daily OME in the 3–12 months before diagnosis. Kaplan–Meier analyses included all MM cases. Multivariable Cox regression was conducted in the subset with available clinical information at diagnosis from the Danish National Multiple Myeloma Register, including ISS stage and MBD status (*n* = 5,494). Information on the ISS stage was determined based on the levels of plasma β2-microglobulin and albumin measured at the time of diagnosis (Supplementary Table 4)^38^, available from the Danish National Multiple Myeloma Register. Cytogenetic/FISH data required for R-ISS or R2-ISS classification were not available within the permissions for the present study. A flowchart of cohort construction is provided in Fig. 3.

### Opioid use

Information on redeemed outpatient opioid prescriptions was obtained from the Danish National Prescription Register using Anatomical Therapeutic Chemical (ATC) codes (Supplementary Table 5).

For the case–control analyses, opioid use was defined for each 3-month interval during the five years before the index date as redemption of ≥ 1 opioid prescription (yes/no). The final 3 -month pre-index interval was excluded as it likely reflects opioids prescribed related to the diagnostic evaluation.

For survival analyses among MM cases, opioid exposure in the 3–12 months before MM diagnosis was quantified using average daily OME. For each redeemed prescription, total OME was calculated from the dispensed amount (strength × quantity) and an opioid-specific conversion factor (Supplementary Table 6)^39,40^. Total OME for the interval was summed across all opioid prescriptions within the interval and divided by the number of days in the interval to obtain average daily OME. Average daily OME was categorized as no use (0 mg/day), low use (< 10 mg/day), and high use (≥ 10 mg/day).

### Statistical analysis

All statistical analyses were conducted using R statistical software version 4.3.2^41^. Significance level was defined as *P* < 0.05, and all tests were two-sided.

#### Prediagnostic opioid use

Associations between opioid prescription redemption and subsequent MM diagnosis were estimated using conditional logistic regression accounting for the matching. Time before the index date was divided into 20 consecutive 3-month intervals during the five years preceding the index date. For each interval, opioid exposure was defined as redemption of ≥ 1 opioid prescription (yes/no). Separate models were fitted for each interval to estimate ORs and 95% CIs comparing cases and controls. Models were adjusted for calendar year and region of residence (categorical). In opioid-type–specific analyses, exposure was defined as redemption of ≥ 1 prescription for the specified opioid during the interval, irrespective of other opioid use. Raw P-values were obtained from each interval-specific conditional logistic regression model and adjusted using the Benjamini-Hochberg FDR procedure within each opioid category across the 19 reported 3-month intervals. As a homogeneity analysis, Breslow-Day tests were used to assess heterogeneity between selected adjacent interval-specific ORs for any opioid use. These analyses were based on crude 2 × 2 tables of case/control status and opioid prescription redemption for the selected adjacent intervals.

#### Survival after MM diagnosis

OS was assessed from the date of MM diagnosis until death, emigration, or end of follow-up, whichever occurred first. Opioid exposure was quantified as average daily OME in the 3–12 months before MM diagnosis and categorized as 0 mg/day, < 10 mg/day, and ≥ 10 mg/day. Kaplan–Meier curves were used to describe OS in all MM cases and compared using the log-rank test. Univariable and multivariable Cox proportional hazards regression models were used to estimate crude and adjusted HRs and 95% CIs. Multivariable models were adjusted for sex, age at diagnosis (continuous), region of residence (categorical), calendar year of diagnosis (continuous), ISS stage (I/II/III), and MBD at diagnosis (yes/no). The multivariable Cox model was restricted to individuals with complete data on covariates.

### Ethics statement

Consistent with the EU General Data Protection Regulation (Article 30), this project was registered in the Danish Cancer Society’s record of processing activities for research projects (2021-CDRC-0033). In accordance with Danish legislation, ethics approval and informed consent are not required for studies based only on register data.

## Supplementary Information

Supplementary Material 1

## Data Availability

The datasets supporting the findings of this study are available from Statistics Denmark and the Danish Clinical Quality Program – National Clinical Registries (RKKP), subject to the necessary approvals. In compliance with Danish legislation, the authors are not permitted to share the data directly. Part of data since 2002 (except for data on healthy matched controls) may be shared on a collaborative basis via access to the Danish Lymphoid Cancer Research (DALY-CARE) data resource upon reasonable request to the corresponding author.
