# Supplementary material for: Prediagnostic opioid use and survival in multiple myeloma: a nationwide register-based study
Source: Sci Rep. 2026 Jul 9;16:31570. doi: 10.1038/s41598-026-59999-2 (PMC13649591; doi:10.1038/s41598-026-59999-2)
Supplement: Supplementary file 1 [file 41598_2026_59999_MOESM1_ESM.docx]

# Supplementary Information for: *Prediagnostic opioid use and survival in multiple myeloma: A nationwide register-based study*

## Supplementary Table 1

**Odds ratios (ORs) for multiple myeloma diagnosis associated with opioid prescription redemption in the interval (yes/no) in consecutive 3-month intervals during the five years before the index date, overall and by opioid category. The interval immediately preceding the index date (0–3 months pre-index; shown as 0.25 years) was excluded a priori because opioid prescribing in this period is likely to reflect peri-diagnostic prescribing related to the diagnostic evaluation; therefore, ORs are not reported for this interval. ORs and two-sided P-values were obtained from conditional logistic regression models. P-values were adjusted for multiple testing using the Benjamini-Hochberg false discovery rate (FDR) procedure within each opioid category across the 19 reported 3-month intervals.**

| **Years before diagnosis** | **Any opioid** | | **Codeine** | | **Tramadol** | | **Morphine** | | **Oxycodone** | | **Buprenorphine** | | **Fentanyl** | |
| --- | --- | --- | --- | --- | --- | --- | --- | --- | --- | --- | --- | --- | --- | --- |
|  | **OR**  **(95% CI)** | **P^1^** | **OR**  **(95% CI)** | **P^1^** | **OR**  **(95% CI)** | **P^1^** | **OR**  **(95% CI)** | **P^1^** | **OR**  **(95% CI)** | **P^1^** | **OR**  **(95% CI)** | **P^1^** | **OR**  **(95% CI)** | **P^1^** |
| 0.25 | - | - | - | - | - | - | - | - | - | - | - | - | - | - |
| 0.50 | 2.42  (2.25-2.60) | < 0.001 | 2.05  (1.74-2.41) | < 0.001 | 2.73  (2.51-2.98) | < 0.001 | 2.59  (2.19-3.08) | < 0.001 | 2.01  (1.66-2.44) | < 0.001 | 2.19  (1.66-2.91) | < 0.001 | 1.86  (1.30-2.65) | 0.004 |
| 0.75 | 1.71  (1.58-1.85) | < 0.001 | 1.74  (1.46-2.07) | < 0.001 | 1.87  (1.69-2.06) | < 0.001 | 1.53  (1.23-1.89) | 0.001 | 1.51  (1.21-1.89) | 0.002 | 1.44  (1.02-2.02) | 0.236 | 2.03  (1.42-2.89) | 0.001 |
| 1.00 | 1.39  (1.28-1.52) | < 0.001 | 1.61  (1.35-1.92) | < 0.001 | 1.44  (1.29-1.60) | < 0.001 | 1.21  (0.96-1.54) | 0.216 | 1.23  (0.96-1.58) | 0.328 | 1.10  (0.75-1.63) | 0.872 | 1.58  (1.04-2.40) | 0.166 |
| 1.25 | 1.33  (1.21-1.45) | < 0.001 | 1.54  (1.29-1.85) | < 0.001 | 1.39  (1.25-1.56) | < 0.001^8^ | 1.21  (0.95-1.54) | 0.216 | 1.25  (0.97-1.60) | 0.328 | 0.97  (0.63-1.48) | 0.928 | 1.39  (0.88-2.21) | 0.622 |
| 1.50 | 1.23  (1.12-1.35) | < 0.001 | 1.44  (1.19-1.74) | 0.001 | 1.28  (1.14-1.44) | < 0.001 | 1.25  (0.98-1.59) | 0.200 | 1.19  (0.92-1.56) | 0.523 | 0.88  (0.56-1.38) | 0.872 | 1.35  (0.83-2.21) | 0.622 |
| 1.75 | 1.13  (1.03-1.24) | 0.032 | 1.51  (1.25-1.82) | < 0.001 | 1.06  (0.94-1.21) | 0.394 | 1.22  (0.96-1.56) | 0.216 | 1.15  (0.87-1.51) | 0.604 | 0.85  (0.53-1.35) | 0.872 | 1.27  (0.77-2.09) | 0.690 |
| 2.00 | 1.10  (1.00-1.22) | 0.100 | 1.21 (0.99,1.49) | 0.106 | 1.15  (1.02-1.29) | 0.065 | 1.03  (0.78-1.34) | 0.879 | 1.05  (0.78-1.41) | 0.797 | 0.72  (0.43-1.21) | 0.714 | 1.09  (0.63-1.88) | 0.872 |
| 2.25 | 1.15  (1.04-1.26) | 0.014 | 1.35  (1.11-1.64) | 0.006 | 1.15  (1.02-1.29) | 0.065 | 1.04  (0.78-1.37) | 0.879 | 1.09  (0.82-1.47) | 0.718 | 1.00  (0.64-1.56) | 0.997 | 1.15  (0.65-2.03) | 0.872 |
| 2.50 | 1.04  (0.94-1.15) | 0.580 | 1.08  (0.87-1.35) | 0.483 | 1.07  (0.94-1.22) | 0.374 | 0.93  (0.69-1.25) | 0.755 | 1.20  (0.90-1.59) | 0.523 | 0.83  (0.51-1.36) | 0.872 | 1.10  (0.61-1.99) | 0.872 |
| 2.75 | 1.01  (0.91-1.12) | 0.942 | 1.01  (0.81-1.26) | 0.909 | 1.09  (0.96-1.24) | 0.294 | 0.87  (0.64-1.18) | 0.496 | 0.89  (0.65-1.24) | 0.718 | 0.92  (0.57-1.48) | 0.900 | 1.16  (0.63-2.16) | 0.872 |
| 3.00 | 1.05  (0.95-1.16) | 0.548 | 1.20  (0.97-1.48) | 0.133 | 1.12  (0.99-1.27) | 0.132 | 0.77  (0.55-1.06) | 0.216 | 0.93  (0.67-1.29) | 0.742 | 0.65  (0.36-1.16) | 0.665 | 1.09  (0.59-2.01) | 0.872 |
| 3.25 | 1.01  (0.91-1.12) | 0.942 | 1.14  (0.92-1.41) | 0.289 | 1.08  (0.95-1.23) | 0.338 | 0.78  (0.56-1.09) | 0.235 | 0.88  (0.63-1.23) | 0.693 | 0.66  (0.37-1.18) | 0.665 | 1.05  (0.53-2.07) | 0.895 |
| 3.50 | 1.10  (0.99-1.22) | 0.119 | 1.42  (1.16-1.72) | 0.001 | 1.14  (1.00-1.29) | 0.092 | 0.69  (0.48-0.98) | 0.155 | 0.69  (0.47-1.01) | 0.287 | 0.77  (0.44-1.35) | 0.872 | 0.95  (0.46-1.94) | 0.895 |
| 3.75 | 1.06  (0.96-1.18) | 0.463 | 1.17  (0.94-1.45) | 0.207 | 1.11  (0.97-1.26) | 0.212 | 0.97  (0.71-1.34) | 0.879 | 0.92  (0.65-1.30) | 0.742 | 0.75  (0.43-1.30) | 0.872 | 0.86  (0.40-1.86) | 0.872 |
| 4.00 | 1.00  (0.90-1.12) | 0.942 | 1.18  (0.95-1.46) | 0.181 | 1.03  (0.91-1.18) | 0.674 | 0.89  (0.64-1.24) | 0.615 | 0.83  (0.58-1.20) | 0.604 | 0.80  (0.46-1.41) | 0.872 | 0.64  (0.26-1.58) | 0.690 |
| 4.25 | 0.99  (0.89-1.10) | 0.942 | 1.09  (0.88-1.37) | 0.473 | 1.01  (0.88-1.16) | 0.918 | 0.65  (0.44-0.96) | 0.146 | 0.80  (0.55-1.17) | 0.554 | 0.95  (0.55-1.63) | 0.928 | 0.69  (0.28-1.72) | 0.788 |
| 4.50 | 1.03  (0.92-1.14) | 0.810 | 1.28  (1.04-1.58) | 0.036 | 1.00  (0.87-1.15) | 0.988 | 0.77  (0.53-1.10) | 0.235 | 0.89  (0.62-1.30) | 0.718 | 0.90  (0.51-1.58) | 0.900 | 0.79  (0.32-1.94) | 0.872 |
| 4.75 | 1.06  (0.95-1.18) | 0.508 | 1.28  (1.04-1.57) | 0.039 | 1.06  (0.92-1.21) | 0.477 | 0.68  (0.46-1.01) | 0.180 | 0.85  (0.58-1.24) | 0.656 | 0.83  (0.46-1.49) | 0.872 | 0.57  (0.21-1.56) | 0.690 |
| 5.00 | 1.03  (0.92-1.15) | 0.810 | 1.09  (0.87-1.36) | 0.483 | 1.09  (0.95-1.25) | 0.309 | 0.76  (0.52-1.12) | 0.235 | 0.99  (0.68-1.43) | 0.954 | 0.95  (0.54-1.68) | 0.928 | 0.48  (0.15-1.52) | 0.622 |

*OR, Odds ratio*

*CI, Confidence interval*

*^1^Benjamini-Hochberg false discovery rate adjusted P-values*

## Supplementary Table 2

**Breslow-Day tests comparing selected adjacent interval specific odds ratios for any opioid use.**

| **Comparison of intervals before index date** | **Breslow-Day χ^2^** | **df** | **P** |
| --- | --- | --- | --- |
| **5.00 vs. 4.75 years** | 0.155 | 1 | 0.694 |
| **4.75 vs 4.50 years** | 0.144 | 1 | 0.704 |
| **4.50 vs 4.25 years** | 0.173 | 1 | 0.678 |
| **4.25 vs 4.00 years** | 0.017 | 1 | 0.897 |
| **4.00 vs. 3.75 years** | 0.534 | 1 | 0.465 |
| **3.75 vs 3.50 years** | 0.270 | 1 | 0.604 |
| **3.50 vs 3.25 years** | 1.386 | 1 | 0.239 |
| **3.25 vs 3.00 years** | 0.263 | 1 | 0.608 |
| **3.00 vs 2.75 years** | 0.278 | 1 | 0.598 |
| **2.75 vs 2.50 years** | 0.206 | 1 | 0.650 |
| **2.50 vs 2.25 years** | 1.761 | 1 | 0.185 |
| **2.25 vs 2.00 years** | 0.334 | 1 | 0.564 |
| **2.00 vs 1.75 years** | 0.097 | 1 | 0.756 |
| **1.75 vs 1.50 years** | 1.551 | 1 | 0.21 |
| **1.50 vs 1.25 years** | 1.345 | 1 | 0.246 |
| **1.25 vs 1.00 years** | 0.631 | 1 | 0.427 |
| **1.00 vs 0.75 years** | 11.085 | 1 | 0.001 |
| **0.75 vs 0.50 years** | 39.841 | 1 | < 0.001 |

## Supplementary Table 3a

**Survival after diagnosis for patients diagnosed with multiple myeloma, stratified by average daily oral morphine equivalent use in the 12 to 3 months before diagnosis (n=6,953).**

| **Average daily OME in the 12 to 3 months before MM diagnosis** | **1-year survival (95% CI)** | **2-year survival (95% CI)** | **3-year survival (95% CI)** | **4-year survival (95% CI)** | **5-year survival (95% CI)** | **P^1^** |
| --- | --- | --- | --- | --- | --- | --- |
| **0 mg** | 82% (81%,83%) | 73% (71%,74%) | 64% (63%,66%) | 57% (55%,58%) | 50% (48%,51%) | < 0.001 |
| **< 10 mg** | 77% (74%,79%) | 66% (63%,69%) | 56% (53%,59%) | 47% (44%,50%) | 40% (37%,43%) |  |
| **≥ 10 mg** | 72% (68%,77%) | 62% (57%,67%) | 53% (48%,58%) | 45% (40%,50%) | 36% (31%,41%) |  |

*CI, Confidence Intervals
OME, Oral Morphine Equivalents
MM, Multiple Myeloma*

^1^ Log-rank test

## Supplementary Table 3b

**Pairwise log-rank tests comparing overall survival between average daily oral morphine equivalent use categories in the 12 to 3 months before diagnosis among patients diagnosed with multiple myeloma.**

| **Comparison** | **Pairwise log-rank P** |
| --- | --- |
| **0 mg vs < 10 mg** | < 0.001 |
| **0 mg vs ≥ 10mg** | < 0.001 |
| **<10 mg vs ≥ 10 mg** | 0.087 |

## Supplementary Table 4

**Criteria for international staging system (ISS) stage used in the study.**

| **Stage** | **Criteria** |
| --- | --- |
| I | Serum β_2_-microglobulin < 3.5 mg/L Serum albumin ≥ 3.5 g/dL |
| II | Not stage I or III |
| III | Serum β_2_-microglobulin ≥ 5.5 mg/L |

## Supplementary Table 5

**Anatomical Therapeutic Chemical (ATC) codes used to define opioid exposure.
Redeemed outpatient prescriptions were identified using the ATC codes listed. Hydromorphone (N02AA03) was excluded due to low use in Denmark.**

| **ATC** | **Opioid** |
| --- | --- |
| R05DA04 | Codeine |
| N02AX02 | Tramadol |
| N02AA01 | Morphine |
| N02AA05 | Oxycodone |
| N02AE01 | Buprenorphine |
| N02AB03 | Fentanyl |

## Supplementary Table 6

**Oral morphine equivalent conversion factors used in the study.**

| **Opioid** | **Route** | **Unit** | **Conversion factor** |
| --- | --- | --- | --- |
| **Codeine** |  |  |  |
|  | Oral | mg | 0.1 |
| **Tramadol** |  |  |  |
|  | Oral | mg | 0.2 |
|  | Parenteral^1^ | mg | 0.2 |
| **Morphine** |  |  |  |
|  | Oral | mg | 1 |
|  | Parenteral | mg | 3 |
|  | Transdermal gel^1^ | mg | 1 |
| **Oxycodone** |  |  |  |
|  | Oral | mg | 1.5 |
|  | Parentheral | mg | 3 |
| **Buprenorphine** |  |  |  |
|  | Buccal/sublingual | mg | 38.8 |
|  | Parenteral | mg | 75.0 |
|  | Transdermal patch^2^ | mcg/hr | 2.2 |
| **Fentanyl** |  |  |  |
|  | Buccal/sublingual | mcg | 0.1 |
|  | Nasal spray | mcg | 0.16 |
|  | Parenteral | mcg | 0.2 |
|  | Transdermal patch^2^ | mcg/hr | 2.7 |

^1^When route-specific conversion factors were unavailable, the oral conversion factor was applied.

^2^For transdermal patches, the prescribed rate (mcg/hour) was converted assuming continuous use over 72 hours (3 days); the conversion factor therefore reflects a full patch-wear period.
